# Hot Transcriptomics

**DOI:** 10.1155/2010/897585

**Published:** 2011-02-07

**Authors:** Jasper Walther, Pawel Sierocinski, John van der Oost

**Affiliations:** Laboratory of Microbiology, Wageningen University, Dreijenplein 10, 6703 HB Wageningen, The Netherlands

## Abstract

DNA microarray technology allows for a quick and easy comparison of complete transcriptomes, resulting in improved molecular insight in fluctuations of gene expression. After emergence of the microarray technology about a decade ago, the technique has now matured and has become routine in many molecular biology laboratories. Numerous studies have been performed that have provided global transcription patterns of many organisms under a wide range of conditions. Initially, implementation of this high-throughput technology has lead to high expectations for ground breaking discoveries. Here an evaluation is performed of the insight that transcriptome analysis has brought about in the field of hyperthermophilic archaea. The examples that will be discussed have been selected on the basis of their impact, in terms of either biological insight or technological progress.

## 1. Thermophiles

Forty years ago it was generally accepted that life was not possible at temperatures higher than 60°C. In 1969, however, Brock and Freeze discovered that the upper temperature limit goes as high as 75°C when microorganisms were isolated from thermal springs in Yellowstone National Park [1, 2]. The pioneering work of Brock set the stage for further exploration of a wide range of thermal ecosystems. Numerous microorganisms defined as thermophiles have since been found to thrive optimally between 50 and 80°C, but also many appeared to have their optimal temperature for growth from 80°C to well above 100°C, the hyperthermophiles. Recently it has been shown that some archaea can endure temperatures as high as 122°C and even proliferate in such conditions. Although there are several bacterial representatives in the group as well, most of the known hyperthermophiles belong to the archaea. 

Thermophilic organisms can be found in water-containing geothermally heated environments. These volcanic ecosystems are mainly situated along terrestrial and submarine fracture zones where tectonic plates are converging or diverging. The terrestrial biotopes of (hyper)thermophiles are mainly aerobic, sulfur containing solfataric fields with temperature as high as 100°C (depending on the altitude) and the pH in a dual range: either acidic (values from below zero to 4.0 [3]) or neutral to slightly alkali (7.0–9.0) [4]. The marine biotopes for (hyper)thermophiles consist of different hydrothermal systems ranging from shallow to abyssal depths. Temperatures in those anaerobic environments can range up to 400°C and the pH is usually in the range of 5.0 to 8.5. 

Progress in culturing thermophilic archaea and in the revolution of DNA sequencing technology has resulted in a rapidly increasing amount of (meta)genomic data on these extreme microorganisms. This has not only led to the discovery of robust biocatalysts but also to fundamental insight into (i) physiology: including unique metabolic enzymes, pathways, and regulation [5–7], (ii) biochemistry: the molecular basis of thermostability of biomolecules [8–10], and (iii) phylogeny: theories on the evolution of the eukaryotic cell [11]. 

The first complete genome analysis of an archaeon, *Methanocaldococcus jannaschii* [12], was a big step towards confirmation of the monophyletic position of the archaea, with respect to the bacteria and the eukaryotes. In addition, archaea appeared to possess a bacterial-like compact chromosomal organization with clustering of genes as polycistronic units (operons), and with only few interrupted genes (introns). Moreover, the archaeal systems that drive the flow of genetic information (transcription, translation, replication, DNA repair) generally correspond to the core of the eukaryal counterparts. These initial observations of bacterial-like “information storage” and eukaryal-like “information processing” have been confirmed by the analyses of subsequently sequenced hyperthermophilic model archaea: the euryarchaea *Pyrococcus* spp. (*P. furiosus, P. abyssi, P. horikoshii*) as well as the crenarchaea *Sulfolobus* spp. (*S. solfataricus, S. tokodaii, S. acidocaldarius*) [6]. The comparative analysis of the genome of the hyperthermophilic bacterium *Thermotoga maritima* to *Pyrococcus furiosus* (both isolated from shallow thermal vents at the same beach (Volcano, Italy)) led to the conclusion that horizontal (or lateral) gene transfer substantially contributes to the apparent high degree of genome flexibility [13, 14]. In addition, the comparison of closely related species (*P. furiosus, P. abyssi, P. horikoshii*) revealed a high degree of genome plasticity. It was also proposed that the lateral gain as well as the loss of genes is a modular event [15]. Horizontal gene transfer has also been proposed to explain the relatively high degree of homology between genomic loci of the euryarchaeon *Thermoplasma acidophilum* and the crenarchaeon* S. solfataricus*, phylogenetically distant archaea, that inhabit the same environmental niche (65–85°C, pH 2.0). The *Sulfolobus*-like genes in the *T. acidophilum *genome are clustered into at least five discrete regions, again indicating modular recombination of larger DNA fragments [16, 17].

After establishing a genome sequence, comparative genomics analyses are performed to assign potential functions for the identified open reading frames. In the majority of the studied prokaryotic genomes, the fraction of hypothetical and conserved hypothetical genes amounts to 40–60% of the coding regions [18]. Hence, one of the main challenges of the postgenome era still is to improve the functional annotation of genes by integrating classical approaches (physiology, biochemistry, and molecular genetics) with genomics-based high-throughput approaches (comparative, functional, and structural genomics). Obvious targets of comparative and functional analysis of archaeal genomes are the numerous missing links in metabolic pathways as well as the largely unknown regulatory systems with either eukaryal or bacterial characteristics [5, 6].

## 2. Archaeal Transcriptomics

DNA microarrays have initially been established as high-throughput functional genomics tools to study eukaryotic and bacterial model systems. Initial assumptions suggested that microarray can be used as a general research tool [19]; however after more than a decade of experience it should be concluded that the application of microarray has its pros and cons. The choice of possible microarray approaches ranges from rather simple layouts comparing two states, to relatively complicated multistate experimental hybridization schemes. The development of appropriate analytical methods has appeared to be a crucial requirement to enable analysis of the more complicated experimental designs and to allow drawing conclusions from relatively small differences in expression profiles. Consequently, high-quality microarray analyses not only require careful experimentation (cultivation, nucleic acid analysis, hybridization) but also state-of-the-art data processing. This has allowed for the high-resolution analysis of time course experiments [20] and of multicondition experiments [21]. In most recent studies, the majority of DNA microarrays are used either (i) as a pilot experiment that should provide leads for further investigations [7], (ii) as a refinement tool to confirm previous gene expression studies [22], or (iii) as one of many high-throughput methods to be integrated in a systems biology analysis [23]. Below, selected examples of transcriptome analyses of (hyper)thermophilic archaea are described in more detail. Selection is has been based on technological and/or scientific impact. An overview of archaeal transcriptome studies can be seen in Table 1.

### 2.1. Sulfur Metabolism

The first microarray analysis reported on either a hyperthermophilic archaeon was a pilot study on *P. furiosus* that focused on a subset of 271 metabolic genes [24]. This analysis focused on a new sulfur-reducing enzyme complex from *P. furiosus*. The experiment showed at least a twofold change in signal intensity for about 50 ORFs that were represented on the array. Subsequently, this initial study was followed by the analyses of a complete genome array [24, 48] using the same strategy. For most genes the complete ORFs were printed on the array as PCR-amplified fragments. These studies addressed the adaptation of *P. furiosus *cells to the availability of sulfur, different carbon sources, and cold shock.

### 2.2. Heat Shock Response

**Figure 1:**
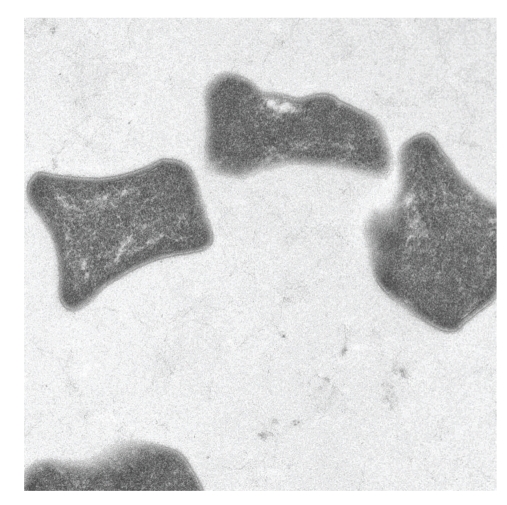
*Sulfolobus solfataricus* cells. Courtesy of Mark Young.

Although hyperthermophiles have a temperature optimum above 80°C, they still can experience heat stress. As in other severe stress conditions, a heat shock will result in retardation or even complete arrest of growth of the organism. This is a consequence of dropping rates of transcription [90]; under such conditions protein synthesis appears to be limited to a subset of proteins that play a crucial role in dealing with the stress factor to allow survival. When a heat shock is experienced by the cell, two of the biggest threats are the denaturation of proteins and the increased fluidity of the membrane. In order to cope with these problems, hyperthermophilic archaea have developed their own strategies to cope with such conditions. The hyperthermophilic heat shock responses of two distinct hyperthermophilic archaea, *P. furiosus *[47] and *S. solfataricus * [59] (Figure 1), were investigated using transcriptomics. Both organisms seem to react to the same kind of stress differently.

The heat shock experiment using *P. furiosus* was conducted by growing the cells on a mixture of tryptone and yeast extract at a suboptimal temperature of 90°C and then shifting the temperature to 105°C [47]. Cells were harvested after 60 minutes and compared to cells grown at 90°C. *P. furiosus* seems to react in several ways: (i) the compatible solutes di-*myo*-inositol-1,1′-phosphate (DIP) and trehalose seem to be produced in order to stabilize its proteins [91]; (ii) proteins were further stabilized by the upregulation of several chaperonin-related genes such as the Hsp60-like thermosome, the Hsp20-like small heat shock protein, and two other proteins (VAT) that are predicted to be involved in both protein unfolding (for proteolyses) and refolding processes; (iii) several genes encoding glycoside hydrolases were upregulated, either as a general stress response or as a directed adaptation to heat stress that may enhance the production of sugar-based compatible solutes. 

The heat shock experiment conducted with *S. solfataricus* was set up differently [59]. The cells were grown at an optimal temperature of 80°C and then shifted to 90°C. Samples were taken 10 minutes before heat shock, 5, 30, and 60 minutes after heat shock allowing for the elucidation of temporal transcriptome changes. This approach showed that about one-third of the genome (~1000 genes) was differentially regulated in the first 5 minutes. Surprisingly, around 200 of the upregulated genes were IS elements, showing that almost all of these selfish elements of *S. solfataricus* are activated when the cells encounter (temperature) stress; it may well be that the transposition by itself also contributes to part of the modulated expression of other genes. In contrast to the findings with *P. furiosus,* no evidence was found of induced expression of enzymes involved in compatible solute production. It has been observed that genes that encode different subunits of the RNA polymerase are downregulated, suggesting that transcription is going down. Furthermore, the gene encoding the DNA polymerase II is down, while several DNA repair-related genes have a higher expression. The expression of several transporter genes (e.g., Iron, Cobalt, Phosphate, Sulfate, Amino Acids, Arabinose, Glucose, Maltose) went down. Interestingly, also many transcriptional regulators were differentially expressed, namely, TetR, and the GntR-like repressors. Furthermore the gene encoding the *γ*-subunit of the thermosome was downregulated, while the genes encoding the *α*- and *β*-subunits were unaffected, which was consistent with the previous findings of a change in composition of the thermosome from 1*α* : 1*β* : 1*γ* to 2*α* : 1*β* : 0*γ* [92]. In conclusion, this experiment showed that in *S. solfataricus* the transcriptional response to a heat shock is instantaneous, but apparently not at the level of compatible solutes. The DNA polymerase II gene is downregulated and a decrease in growth rate is observed. Furthermore the transcription of different subunits of the RNA polymerase is reduced suggesting a global transcription reduction. Many transcriptional regulators appear to play a role in coping with a heat shock in *S. solfataricus,* and it would be very interesting to establish their specific function, that is, their target promoters. The difficulty in comparing these two studies is mainly caused by the different sampling approach. In case of *S. solfataricus *the shift has been made from the temperature at which the growth is the fastest; in case of *Pyrococcus *there might be additional variation in the results related to the suboptimal temperature at the beginning of the experiment.

### 2.3. Viral Infections and Microorganism Interactions

In most environments viral particles significantly outnumber microbial cells, indicating that viral infection is a common threat to the majority of organisms. Hyperthermophiles are not an exception to this rule. Here we discuss two viral infection studies of *S. solfataricus*, both of which have been conducted by using DNA microarrays that contained oligonucleotides corresponding to genes of both *S. solfataricus *as well as genes from selected *S. solfataricus* viruses and plasmids. One study described infection by the lytic virus STIV (*Sulfolobus Turreted Icosahedral Virus) *that usually only kills part of the* S. solfataricus *population in its life cycle [93], whereas comparable analyses have been performed on the well-studied lysogenic SSV1 virus (*Sulfolobus shibatae* Virus 1) [94].

**Figure 2:**
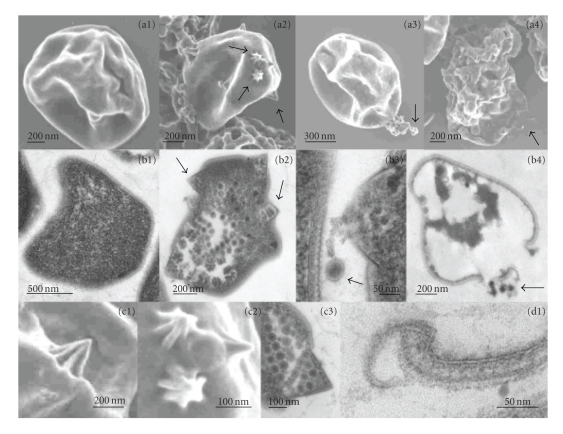
SEM images (row a) and corresponding TEM images (row b) of *S. solfataricus* cells show different stages of infection. (a1 and b1) Noninfected cells. (a2 and b2) Cells infected with STIV displaying membrane protrusions (thin arrows). (a3 and b3) Lysing cells releasing virus (thin arrows) and cell contents. (a4 and b4) Empty cells showing S-layer and broken membrane fragments (thin arrows). Pyramid-like structures from STIV-infected cells observed by SEM (c1 and c2) and TEM (c3) are also shown. (d1) TEM image of broken membrane and S-layer after cell lysis. Scale bars are indicated (courtesy of Mark Young).

The study of STIV conducted by Ortmann et al. [80] comprises of the isolation of a *S. solfataricus *mutant that is hypersensitive to the studied virus with almost all cells of a culture being killed in the lytic cycle. STIV is a dsDNA virus with a circular genome of 17 kb, containing 37 predicted ORFs. Analysis of the viral transcriptome showed the upregulation of 47 of the 52 viral microarray probes, which cover the viral genes and some intergenic regions in both directions. Transcription of viral genes was first detected at 8 hpi (hours post infection), whereas at 16 hpi most viral genes are expressed. At 24 hpi a shift takes place from virus replication to preparation for lysis and around this time point most viral genes are expressed; general cell lysis occurs at 32 hpi. Although the expression starts at different time points, no real temporal expression has been observed in this experiment; however, one cannot rule out that this is a resolution issue due to suboptimal synchronization of the infection cycle. At the early stage of viral gene expression (8 hpi) there are four transcripts and an intergenic region that are being expressed. These genes are most probably responsible for initiation of the early infection process. Expression of most structural viral genes is found at 16 hpi and thereafter. Of the 177 host genes that were differentially regulated (more than 2-fold), of which 124 were upregulated, most are associated with either DNA replication and repair or genes of unknown function, suggesting that STIV uses host proteins to aid the replication of its own DNA. An important upregulated protein concerns the ESCRTIII homolog, which has recently been reported to be essential for the cell division in *Sulfolobales* [95, 96]; the upregulation may suggest involvement in the recently discovered release system for both STIV and SirV that involves unique pyramid-like structures (Figure 2) [97, 98]. All of the downregulated host genes were regulated just before cell lysis at 32 hpi and were associated with metabolism. 

An infection study of SSV1 with *S. solfataricus *as a host has been conducted in order to find out more about the transcriptome fluctuations of this lysogenic virus and its host [99]. Initially infection by SSV1 seems not to affect the growth rate of the infected cells; at least partly, the SSV1 genome is integrated at a specific site in the host chromosome [100]; however, as soon as SSV1 starts to produce and release viral particles, the cell growth is significantly retarded. Viral production can be greatly stimulated after UV induction. The first viral transcripts can already be found at 1 hpi, while most viral genes are active at 8.5 hpi. The viral genes are clustered as 9 operons, comprising both regulatory genes and structural genes. The regulatory genes are the first ones to be transcribed, and the genes coding for the coat protein of the virus are produced at a later stage. 

There are more differences between the two studies, and only few similarities. Comparison of the two datasets is not straightforward, mainly because it compares infection by two distinct types of viruses (lytic versus lysogenic); in addition there are some methodological differences like the different time points involved, number of time points taken into account, and so forth. One of the main differences concerns the fact that STIV seems to have a larger impact on the host due to a more profound regulation of host genes (177 instead of 55); this may correlate with its lytic live-cycle. However, to deduce general patterns it will be necessary to compare the transcription profiles during a synchronized infection of additional viruses. A recent study on the infection of the closely related *S. islandicus* with the lytic virus SirV revealed a dramatic degradation of the host chromosome upon viral assembly and proliferation [98]; no transcriptome analysis of host genes after infection of this system has yet been reported.

The microarray technique can be used to observe the interactions between two distinct species. One such attempt has been done on a bacteria, *Thermotoga maritima*, which has been grown alone as well as in a coculture with a archaea, a methanogenic thermophile, *Methanocaldococcus janaschii* [101]. This experiment yielded an interesting view on the importance of the H_2_ transfer in hot environment. The experiment focused on a shift from the mid logarithmic growth phase to the early stationary. It has been observed that the growth of *T. maritima* has been boosted 3- to 5-fold due to removal of inhibiting H_2_. Also the methane production of *M. jannaschii* has been increased twofold compared with pure culture. The transcriptome analysis of the 2 samples from the early stationary phase showed that in the pure culture of *T. maritima*, 127 genes have been significantly upregulated in comparison with the coculture. Half of those were associated with the central carbon metabolism. At the same time, in the coculture of the 113 genes upregulated, the main groups present were ABC transporters and carbohydrate hydrolases. This suggests that the pure culture conditions support the main metabolic pathways while the coculture conditions seem to boost the scavenging. The scavenging strategy may be boosted by the exopolysaccharide (EPS) produced by the coculture cells that form aggregates to enhance the hydrogen transfer [102]. Another, less obvious conclusion from the experiment was the confirmation that in this case, a microarray platform designed to analyze one species can be successfully used to analyze a coculture condition.

### 2.4. Genome Replication and the Cell Cycle

**Figure 3:**
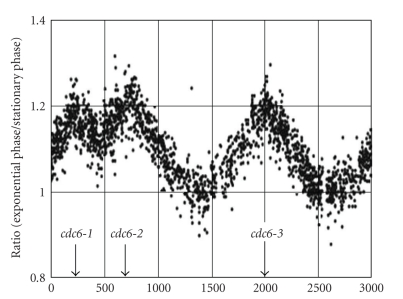
Marker Frequency distributions: exponential growth versus stationary phase for *S. solfataricus* (courtesy of Magnus Lundgren). Here DNA from *S. solfataricus* cells in exponential phase was compared to DNA from cells in stationary phase. Cells that just have begun growing have more copies of genes at or close to a DNA replication site than DNA further from the replication start site. Therefore genes close to a replication start site will have a higher ratio than genes not close to such a site and this is seen as a peak in the figure. The figure has three clear peaks, showing that *S. solfataricus* has 3 origins of replication; each peak is located near a predicted *cdc6* site.

Up until 2004 it was assumed that genome replication with multiple origins of replication was a typical Eukaryotic-like feature [103]. In 2004, different groups independently discovered that *Sulfolobus* spp. has multiple origins of replication [68, 104, 105]. Using 2D DNA gels, two origins of replication could be demonstrated in *S. solfataricus,* while a microarray approach (quantification of genomic DNA by hybridizing it with a DNA microarray) was used to prove that *Sulfolobus* spp. has actually three origins of replication (Figure 3). In the latter study *Sulfolobus* cells were treated with acetic acid in order to synchronize the initiation of replication. After removal of the acetic acid inhibition, the cells were harvested at different time points and genomic DNA was extracted and hybridized on a microarray. It was revealed that all three *cdc6*-like genes in both* S. acidocaldarius* and *S. solfataricus* were functional. Although this was a major breakthrough in the field of prokaryotic genome replication, it should be stressed that other archaea (incl. *P. abyssi*) have a single origin of replication [103]. Together with the fact that none of the known bacterial chromosomes possess multiple origins, this strongly suggests that multiple origins are an archaeal invention, and that the last universal common ancestor (LUCA) most likely possessed a single origin of replication [106, 107].

The cell cycle of the *Sulfolobus* spp. is relatively well studied and, although some archaeal species show modifications to this model [69, 108], it is currently used as archetype of the archaeal cell cycle. An important mechanistic difference, however, concerns the involvement of the ESCRT-III-based system in crenarchaea, versus the FtsZ-based, tubulin-directed system in euryarchaea [109]. *S. solfataricus*, interestingly, possesses both the ESCRT-III encoding genes as well as a gene hypothesized to be an FtsZ paralog [6]. In 2007, Lundgren and Bernander used a microarray approach to analyze a time series of synchronized cells of* S. acidocaldarius* to show that a cyclic induction of genes is involved in the cell cycle [20]. The cell growth was arrested in the G2 phase by addition of acetic acid (dissipates membrane potential and inhibits overall metabolic activity at low pH); after resuspending the cells in fresh medium, the synchronized cells started to grow again after 30 minutes. Cells were analyzed at 8 different time points allowing a good overview of global gene expression patterns starting at the G2 phase (0–30 minutes) going all the way through the cycle until the cells are again in the G2 phase (about 200 minutes later). In a parallel study, using a distinct manner of synchronization in which cells are captured at low temperature right after cell division (the baby machine), Samson et al. presented a cell cycle-dependent transcription of ESCRT-III system components and a Vps4 homolog in *S. acidocaldarius* [110]. Interestingly, though not annotated as ESCRT/Vps4, similar expression profiles of these genes were described in the parallel study mentioned above [111]. The observed activity of ESCRT-III system in Crenarchaeal cell cycle suggests a common ancestry of cell division mechanisms in archaea and eukarya.

Apart from shedding light on the cell division mechanisms, microarray analysis allowed observing a cyclic expression of different kinases, at least seven transcription factors, as well as the three *cdc6 *genes. These findings suggest that the cell cycle is regulated at different levels. Of the three *cdc6 *genes, *cd6-1* is the first to be highly expressed, slightly before the G_1_/S transition. Shortly after the induction of the first *cdc6* gene, the *cdc6-3* gene is induced, confirming its secondary role to the *cdc6-1* gene. The gradual induction of the *cdc6-2* gene slightly before the cells approach the G_2_ phase suggests a negative regulatory role in chromosome regulation as suggested in earlier studies [104]. On the other hand, the data from Duggin et al. [112] implies that the Cdc6 protein levels during the cell cycle synchronized using the baby machine remain unchanged. The discrepancy between the results is hypothesized to be an effect of two different synchronization methods rather than from the cell cycle itself. Acetate can induce stress in the cells and influence transcription of some stress response-related genes. It can also be a result of differential levels of transcript levels and protein; however this possibility is undermined by the fact that other studies showed a correlation between protein and transcript level in case of this gene [23, 57, 113].

### 2.5. Pentose Metabolism in Archaea

Most genomes consist of considerable fractions of hypothetical genes for which a function cannot accurately be predicted. These genes are either too distantly related to well-established orthologs to be recognized as such; alternatively, they may encode novel types of proteins, either involved in unique processes/bioconversions or playing a role in a known process but being the result of a nonorthologous gene displacement [114]. Microarrays can help elucidating the function of these hypothetical genes, by comparing the transcriptomes in condition where a given process/pathway is expected to be active or not. As such, appropriate transcription profiles could serve as leads for further research. 

A good example of a successful microarray-based discovery in archaeal metabolism concerns the elucidation of a pentose-converting pathway in *S. solfataricus*. Unlike many other bacteria and eukaryotes, archaea do not seem to have the classical oxidative pentose phosphate pathway to produce pentose precursors. In addition, until recently the mechanism of the catabolic process of many pentoses in archaea was not understood in great detail [115, 116]. The analysis of Brouns et al. helped to understand how D-arabinose is metabolized by *S. solfataricus*; moreover, insight was gained in the composition of some general pentose oxidation pathways in both archaea and bacteria [7]. In this study, the microarray technology has been used as an initial step of pathway elucidation and allowed for composing a short list of potential candidate enzymes. Comparison between cells grown on D-arabinose and D-glucose revealed that 16 genes were significantly upregulated in the first condition. These included the genes encoding the 4 subunits of a previously identified arabinose ABC transporter, a putative sugar permease, and 5 hypothetical enzymes. Comparing the sequences of the intergenic regions revealed the presence of a conserved palindromic motif in promoter regions of 5 of the upregulated genes: the arabinose ABC transporter operon, and 4 of the hypothetical genes. Production and characterization of the 4 corresponding enzymes has resulted in unraveling the arabinose degrading pathway. 

A further *in silico* investigation of the genes resulted in the finding of different but very similar degradation pathways for several C_5_ (D- and L-arabinose, D-xylose, hydroxyl-proline) and C_6_ (D-glucaric acid, D-galactaric acid) substrates [7], used by different organisms. Interestingly, all proposed pathways converge at 2,5-dioxopentanoic acid, which is converted to the citric acid cycle intermediate 2-oxoglutaric acid (*α*-ketoglutarate). This is yet another example of the metabolic tinkering during the evolution of metabolic pathways [114]. As biochemical pathways of archaea can be very different from their bacterial/eukaryotic counterparts, DNA microarrays in combination with the currently established gene disruption techniques for *Sulfolobus spp.* [117] and *Thermococcus kodakaraensis* [118] may provide a solid basis for subsequent analyses.

## 3. Deep Sequencing: The High-Resolution Alternative

The next generation transcriptomics approach is deep sequencing. In deep-sequencing protocols, RNA is used to generate complementary DNA (cDNA) that will then be sequenced, generating reads of ~400 nucleotides (454/pyrosequencing [119]) and/or reads of ~75 nucleotides (Solexa/SOLiD [120]). A major practical advantage is that this procedure is based on general, species-independent protocols. In addition, it does not need the pre-existing knowledge of the species' genome. Moreover, it allows for comparison of multiple species in coculture by simultaneous analysis using the same platform. Because of these features, this technology frequently used the transcriptomics analysis of environmental samples. 

A disadvantage of this approach for analysis of prokaryotic transcriptomes is the overabundance of the rRNA-species, compared to the mRNA-species (only <5% of the total cellular RNA consists of mRNA). This overabundance of non-mRNA species in the sequenced sample results in a high-noise factor and also could result in not detecting mRNA that is present in only low amounts. Therefore many protocols rely on the specific removal of rRNA before actual sequencing [121]. Most of them are based on techniques that fish out mRNA by using the poly-A tail, which eukarial mRNA posses, but prokaryotes do not. Despite these practical challenges, Wurtzel et al. have successfully analyzed the transcriptome of *S. solfataricus* by deep sequencing, without the removal of the rRNA [83]. They have grown the organism on glucose, cellobiose, and cellulose and sequenced the cDNA using the Illumina Genome Analyzer (Solexa). Of the originally proposed set of 3300 genes [122], the deep-sequencing study managed to correct the annotation of 162 genes, define 80 new ORFs, predict 80 noncoding RNA's, predict a possible hypersensitive RNA cleavage site, and determine the operon structures of more than 1000 transcriptional units. Moreover, they have found that at least 80 of the *S. solfataricus* operons have overlapping antisense transcripts, a relatively high number (8%) in prokaryotes. These *cis*-encoding transcripts most likely play a role in control of gene expression at either transcriptional or translational level [123].

## 4. Standardized Procedures

High-throughput functional genomics approaches are frequently combined in systems biology approaches aiming at modeling the physiology of microbial cells. A very good example of such a systems approach in mesophilic archaea is a study by Bonneau et al. [124], in which transcriptome analysis was part of an integrated analysis aiming at the reconstruction of a gene networks in the halophilic archaeon Halobacterium sp. By using different transcription regulators, genetic modification, and high-throughput methods, a model has been generated that describes the behavior of this network in a range of conditions. Such a systems approach combined with modeling allows picturing the interactions of an organism and predicting its behavior in the natural environment. The difficulty of such an approach lies in synchronizing a large research project and having a uniform biomaterial to start with. 

An example of such a systems biology approach in thermophilic archaea concerns the SulfoSYS project [23], which is part of the European SysMO consortium. A major goal of the latter consortium is to establish well-integrated systems biology projects on selected model organisms. A major goal of the SYSMO projects is to perform a multidisciplinary, functional genomics approach that should be highly reproducible because of the implementation of well-described, standard protocols. In the SulfoSYS project the model organism S. solfataricus is cultivated in a very controlled way. The obtained cells are then distributed among the different researchers to perform transcriptomics, proteomics, metabolomics, as well as biochemical analyses; eventually the data are included in an integrated metabolic model. The stringency of cultivation and sampling has been important also due to a comparison of cells from different temperature values. As the half-lives of some mRNA particles can be as low as 2 minutes [98], a slight difference in sampling may lead to a large difference in the transcript level. The impact of the careful preparation of biological samples in functional genomics analyses, including DNA microarray experiments, has not always been appreciated; on the other hand it is generally accepted that this may significantly affect the reproducibility of this approach. The SulfoSYS project puts much weight on careful sample preparation and on verifying the quality of the obtained cell material before performing actual experiments [125]; this has resulted in a combined dataset with microarray and deep sequencing data that are in very good agreement (Sierocinski et al., unpublished). The SysMO consortium puts extra weight on giving an unrestricted and easy access to the generated data [126]. As far as the datasets of respective microarrays are usually freely available, the multitude of standards, methods, and platforms severely impedes the possibilities of comparing two datasets with each other. Applying the deposition standards, as Minimum Information About a Microarray Experiment (MIAME) [127], certainly helps to validate the quality of the data; however, a simplified standard for results storage could be proposed to allow quick and efficient analysis of deposited datasets.

## 5. Conclusions and Outlook

DNA microarrays have been very successful during the last decade, as a high-throughput research tool that has led to important scientific discoveries, including important findings on cell biological/metabolic features of hyperthermophilic archaea, as outlined above. The most frequently used DNA microarrays (based on oligonucleotides) have restrictions because the probe design is based on previously made assumptions with respect to predicted genes; this implies that small ORFs and noncoding RNAs are generally not included on microarrays. In addition, the commonly used technology only allows for relatively limited numbers of spots that can be printed on one slide. The problem of an incomplete set of probes is solved by using tiled DNA microarrays, which are composed of overlapping oligonucleotides. The used probe lengths and the degree of tiling between overlapping probes determine the resolution that can be achieved; typically 2–4 × 10^5^ probes are printed per slide, with probe size ranging between 50 and 75 nucleotides. Tiled arrays cover the two complete strands of the target chromosomes [128]. 

New ways of obtaining global transcriptomic data are being investigated. Sequencing cDNA (RNA-seq), although still a developing technique, seems to be very promising [129]. This approach is easier to implement for eukaryotic systems, due to the polyA-based procedure for separating mRNA from the contaminating rRNA. However, despite this practical complication, this technology will also be an important step forward in the transcriptome analysis in prokaryotic systems. In eukaryotes ORF prediction is not as easy as in prokaryotes and this has often led to the development of cDNA libraries for the production of microarrays. RNA-seq, although frequently used in eukaryotic transcriptomics, might become of more importance in future transcriptome studies of bacteria and archaea. Recently some groups have started to gain insight into the expression levels of the complete transcriptome using high-throughput sequencing techniques like 454 deep sequencing [121]. Reads of 400 bps can be obtained, at a cost which almost equals the cost for microarray hybridization, with a 97% certainty of prediction of the messenger RNA species [130, 131]. This sequencing approach has the advantage that the same platform can be used for different species, resulting in a better interspecies comparison by omitting the cross-platform bias. This opens up the door for environmental transcriptome profiles, allowing for the monitoring of metagenome-based gene expression in the environment, as opposed to the artificial conditions that are generally imposed on them in a laboratory setting. A further advantage might be that RNA-seq is less prone to signal loss due to mutations that arise during cultivation. Although this technique is not yet readily accessible for most labs, the anticipated reduction of sequencing costs in the near future might make this a very attractive general technique for transcriptome analysis for both eukaryotes and prokaryotes. A decrease in the use of the DNA microarray as a research tool and an increase of using sequencing-related techniques in this field may be expected [132]. 

RNA-seq might turn out to be quintessential in examining environmental samples where not all of the components have been known beforehand. For instance, they might greatly help to increase our understanding of phage pressure on the potential hosts that takes place in situ by finding more viral transcripts and watching the response of the thermophiles to multiple viruses present in the environment. One can assume that hyperthermophilic environments are a very good target for early attempts of metatranscriptomic analyses as the ecology of such niches is generally less complex than that of aquatic or soil ecosystems, making it easier to deal with big dataset covering many organisms.

## Figures and Tables

**Table 1:** A list of different archaeal transcriptome publications. This table shows that transcriptome studies are mostly done to elucidate metabolic processes or the behaviour of different archaea in stress situations. The publications are sorted by subject. Per subject the publications are sorted by year of publication. We included some environmental studies because they give a crucial insight in the ecological function of archaeal species. We excluded some of these publications because in our view they focused more on nonarchaeal species, which is a subject not related to this article. The studies referring to thermophiles are in bold. The studies more described in this paper in more detail are marked with an asterisk next to the reference.

Organism	Subject studied	Reference
	**Metabolism**	

***Pyrococcus furiosus***	**Sulfur metabolism**	[24]*
*Halobacterium salinarum NRC-1*	Adaptation to phototrophy	[25]
*Haloferax volcanii*	Central carbon metabolism	[26]
***Pyrococcus furiosus***	**Central carbon metabolism**	[27]*
*Halobacterium salinarum NRC-1*	Anaerobic respiration	[28]
*Methanosarcina mazei*	Metabolism of methanogenic substrates	[29]
***Sulfolobus solfataricus***	**Central carbon metabolism**	** [30]**
***Sulfolobus solfataricus***	**Pentose metabolism**	[7]*
*Methanosarcina barkeri*	Methanogen metabolism/methods	[31]
*Methanosarcina mazei*	Nitrogen metabolism and regulation	[32]
***Pyrococcus furiosus***	**Starch metabolism**	** [33]**
***Pyrococcus furiosus***	**Metabolism of elemental sulfur**	** [34]**
*Halobacterium salinarum R1*	Adaptation to phototrophy	[35]
*Methanosarcina acitovorans*	Acetate and methanol metabolism	[36]
Environmental array	Ammonium oxidation	[37]
***Metallosphaera sedula***	**Electron transport chain**	**[38]**
*Methanosarcina *	Methanogenesis	[39]
***Pyrobaculum aerophilum***	**Terminal electron acceptor studies**	**[40]**
***Thermoproteus tenax***	**Central carbohydrate metabolism**	**[41]**
*Halobacterium salinarum R1*	Phosphate-dependent behaviour	[42]
*Halobacterium salinarum NRC-1*	Global response to nutrient availability	[43]
*Haloferax volcanii*	D-Xylose metabolism	[44]
*Methanosarcina mazei*	Response to nitrogen availability	[45]
***Metallosphaera sedula***	**Auto- hetero- and mixotrophic growth**	** [21]**
***Metallosphaera sedula***	**Bioleaching**	**[46]**

	**Stress**	

***Pyrococcus furiosus***	**Heat shock response**	[47]*
***Pyrococcus furiosus***	**Cold shock response**	**[48]**
*Halobacterium salinarum NRC-1*	UV irradiation	[49]
***Methanocaldococcus janaschii***	**Heat and cold shock**	**[50]**
*Methanosarcina barkeri*	Heat shock and air exposure	[51]
***Methanocaldococcus janaschii***	**Pressure stress**	**[52]**
***Pyrococcus furiosus***	**Response to gamma irradiation**	** [53]**
*Methanosarcina mazei*	Salt adaptation	[54]
*Methanococcus maripaludis*	H-limitation and growth rate	[55]
*Halobacterium salinarum NRC-1*	Response to change in temperature and salinity	[56]
***Sulfolobus solfataricus***	**UV irradiation**	**[57]**
***Sulfolobus solfataricus; S. acidocaldarius***	**UV irradiation**	**[58]**
***Sulfolobus solfataricus***	**Heat Shock Response**	[59]*
*Halobacterium salinarumNRC-1*	UV irradiation	[25]
***Sulfolobus solfataricus***	**Oxygen stress**	**[60]**
***Sulfolobus solfataricus***	**Oxygen stress**	**[61]**
*Methanococcoides burtonii*	Heat stress	[62]
***Thermococcus kodakaraensis***	**Heat stress**	** [63]**
***Pyrococcus furiosus***	**Heat stress**	** [64]**
***Sulfolobus solfataricus***	**Heat stress**	**[65]**
***Pyrococcus furiosus***	**Oxidative stress**	** [66]**
*Methanohalophilus portucalensis*	Hypo- and Hyper-salt stress	[67]

	**Replication**	

***Sulfolobus solfataricus; S. acidocaldarius***	**Origin of replication**	[68]*
*Halobacterium salinarum NRC-1*	Cell cycle regulation	[69]
***Pyrococcus abyssi***	**Origin of replication**	**[70]**
***Sulfolobus acidocaldarius***	**Cell cycle**	[20]*

	**Various**	

Environmental array	Methanotroph diversity in landfills	[71]
**Pyrococci**	**Genomic DNA hybridization**	**[72]**
***Sulfolobus solfataricus; S. acidocaldarius***	**RNA decay**	**[73]**
*Methanococcus maripaludis*	Mutant studies	[74]
*Haloferax volcanii*	Promoter studies	[75]
***Thermococcus kodakaraensis***	**Promotor studies**	** [76]**
***Thermococcus kodakaraensis***	**Archaeal operon prediction**	**[77]**
*Haloferax volcanii*	Deletion mutant analysis	[78]
Environmental array	Detection of acidophilic activity	[79]
***Sulfolobus solfataricus***	**Viral infection**	[80]*
***Sulfolobus***	**Genomic hybridizations**	**[81]**
***Sulfolobus***	**Transcription bias near OriC**	**[82]**
***Sulfolobus solfataricus***	**Single base resolution map of the genome**	[83]*
Environmental array	Antarctic soil community	[84]
*0Methanosarcina acetivorans*	Regulation of genes	[85]
*Halobacterium salinarum *R1	Control of multiple genes by regulatory proteins	[86]
*Haloacterium salinarum *NRC-1	Physiological readjustments during growth	[87]
Environmental array	Methanogens in cattle excreta	[88]
Environmental array	Gene transfer	[89]
